# Dynamic expression of viral and cellular microRNAs in infectious mononucleosis caused by primary Epstein-Barr virus infection in children

**DOI:** 10.1186/s12985-015-0441-y

**Published:** 2015-12-03

**Authors:** Liwei Gao, Junhong Ai, Zhengde Xie, Chen Zhou, Chunyan Liu, Hui Zhang, Kunling Shen

**Affiliations:** Key Laboratory of Major Diseases in Children and National Key Discipline of Pediatrics (Capital Medical University), Ministry of Education, Beijing Pediatric Research Institute, Beijing Children’s Hospital, Capital Medical University, Beijing, 100045 China; Department of Respiratory, Beijing Children’s Hospital, Capital Medical University, Beijing, 100045 China

**Keywords:** Epstein-Barr virus, Infectious mononucleosis, microRNA, Children

## Abstract

**Background:**

Epstein-Barr virus (EBV) was the first virus identified to encode microRNAs (miRNAs). Both of viral and human cellular miRNAs are important in EBV infection. However, the dynamic expression profile of miRNAs during primary EBV infection was unknown. This study aimed to investigate the dynamic expression profile of viral and cellular miRNAs in infectious mononucleosis (IM) caused by primary EBV infection.

**Methods:**

The levels of viral and cellular miRNAs were measured in fifteen pediatric IM patients at three different time-points. Fifteen healthy children who were seropositive for EBV were enrolled in the control group. Relative expression levels of miRNAs were detected by quantitative real-time PCR (qPCR) assay.

**Results:**

EBV-miR-BHRF1-1, 1-2-3P, miR-BART13-1, 19-3p, 11-3P, 12–1, and 16–1 in IM patients of early phase were significantly higher than in healthy children. Most cellular miRNAs of B cells, such as hsa-miR-155-5p, −34a-5p, −18b-5p, −181a-5p, and −142-5p were up-regulated; while most of cellular miRNAs of CD8 + T cells, such as hsa-miR-223, −29c-3p, −181a, −200a-3p, miR-155-5p, −146a, and −142-5p were down-regulated in IM patients. With disease progression, nearly all of EBV-miRNAs decreased, especially miR-BHRF1, but at a slower rate than EBV DNA loads. Most of the cellular miRNAs of B cells, including hsa-miR-134-5p, −18b-5p, −34a-5p, and -196a-5p increased with time. However, most of the cellular miRNAs of CD8 + T cells, including hsa-let-7a-5p, −142-3p, −142-5p, and −155-5p decreased with time. Additionally, hsa-miR-155-5p of B cells and hsa-miR-18b-5p of CD8+ T cells exhibited a positive correlation with miR-BHRF1-2-5P and miR-BART2-5P (0.96 ≤ r ≤ 0.99, *P* < 0.05). Finally, hsa-miR-181a-5p of B cells had positive correlation with miR-BART4-3p, 4-5P, 16–1, and 22 (0.97 ≤ r ≤ 0.99, *P* < 0.05).

**Conclusions:**

Our study is the first to describe the expression profile of viral and cellular miRNAs in IM caused by primary EBV infection. These results might be the basis of investigating the pathogenic mechanism of EBV-related diseases and bring new insights into their diagnosis and treatment.

## Background

Epstein-Barr virus (EBV), an oncogenic virus of gamma herpes family, infects over 95 % of people worldwide. B lymphocytes are the target cells in primary EBV infection. Most children with primary EBV infection are asymptomatic, but some children can manifest as infectious mononucleosis (IM). IM is a self-limited disease which is due to the transient proliferation of EBV-infected B cells accompanied by excessive response of EBV-specific cytotoxic T cells (CTL) [1]. A previous study [2] showed a positive correlation between the levels of CD8+ T cells and NK cells and the severity of IM. After acute phase, EBV drives the proliferation and differentiation of the naïve B cells into memory B cells and persists for the life of the host in a latent state in the memory B cells. This could allow EBV to escape immune recognition and prevent immune-mediated destruction by the host. However, when the cellular immune function is disturbed, EBV can cause lymphoproliferative diseases, such as lymphomas.

Small non-coding microRNAs (miRNAs) are key cellular regulatory elements and play critical roles in a variety of cell signaling pathways [3]. MiRNAs are single-stranded RNAs (ssRNAs) (approximately 18–25 nucleotides) which regulate gene expression at the post-transcriptional level by base pairing with target mRNAs, leading to mRNA cleavage or translational repression. EBV was the first virus demonstrated to encode miRNAs, including 44 mature miRNAs encoded by two primary transcripts (BHRF1 and BART) [4]. Many studies [5] have demonstrated that EBV-miRNAs target viral and cellular genes which influence the immune response and contribute to EBV-induced malignancy. For example, miR-BHRF1-3 [6] targets the interferon (IFN)-inducible T-cell-attracting chemokine (CXCL11) which contributes to the survival of infected cells. Nachmani et al. [7] also reported that miR-BART2-5p could target MICB, a stress-induced natural killer (NK) cell ligand, and allowed EBV-infected cells to escape recognition. EBV infection can also alter the expression of cellular miRNAs during EBV infection which are capable of regulating the immune response, apoptosis, proliferation, and cellular differentiation [8]. Some studies [9] have found that miR-155 and miR-21 of B cells were induced in EBV infection, thus potentially play a role in viral tumorigenesis.

Nevertheless, EBV and cellular miRNAs are differentially expressed in many diseases [10, 11]. A previous study [12] showed that BHRF1 miRNAs were expressed at high levels in cells of type III EBV latency (such as IM), but lower in type I and II EBV latency. While BART miRNAs were observed in all latent infection types and expressed at high levels in type II EBV latency, such as nasopharyngeal carcinoma (NPC). Cameron et al. [13] demonstrated that the levels of has-miR-21, miR-23a, miR-24, miR-27a, miR-34a, miR-146a/b, and miR-155 were higher in type III EBV latency cells than in type I latency cells. Whereas, miR-28 expression was lower in type III latency. For instance, in Hodgkin’s lymphoma, hsa-miR-155, −21, −20a, −9, −16, and −429, were up-regulated, while BHRF1 miRNAs and hsa-miR-200a were down-regulated [14, 15]. Determining the expression profile and dynamic changes of viral and cellular miRNAs in IM with primary EBV infection is a fundamental and important aspect in investigating the pathogenic mechanism of EBV-associated diseases. However, relatively little is known about the dynamic expression of miRNAs during primary EBV infection. To address this gap, ours’ is the first study that describes the dynamic expression of viral and cellular miRNAs in IM caused by primary EBV infection.

## Results

### Levels of viral loads in plasma and PMBCs

**Fig. 1:**
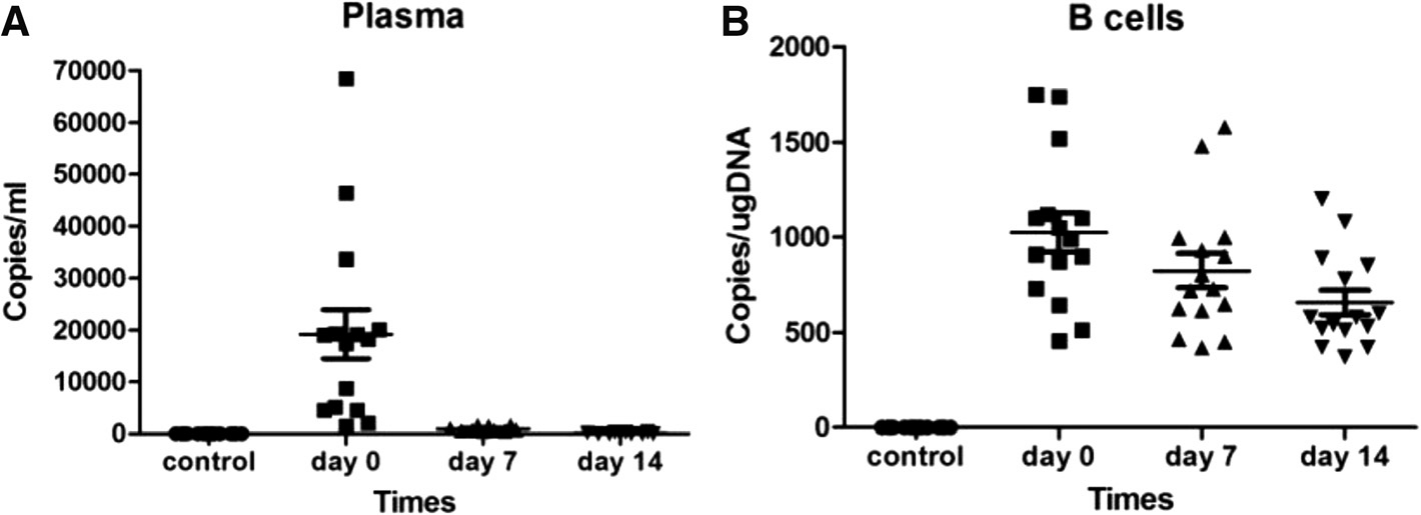
Kinetic analysis of viral loads in patients with IM at day 0 (n = 15), day 7 (n = 15), and day 14 (n = 15). **a** The levels of viral loads in plasma at different time-points. **b** The levels of viral loads in B cells at different time-points

The viral loads in plasma and PBMC of patients were detected at days 0, 7, and 14 respectively. In plasma, the average viral loads at days 0, 7, and 14 were 1.92 × 10^4^ copies/mL (range 1.52 × 10^3^-6.84 × 10^4^ copies/mL), 9.63 × 10^2^ copies/mL (range 5.2 × 10^2^-1.63 × 10^3^ copies/mL), and negative for all, respectively. Viral loads of plasma decreased sharply and were not detected at day 14 (Fig. 1a). In detecting the viral loads in four types of lymphocytes at day 0, B cells showed the highest viral loads (median 1.02 × 10^3^ copies/μg DNA, range 5.13 × 10^2^-1.75 × 10^3^ copies/μg DNA) while there were no detectable viral loads in the other lymphocyte subpopulations. This indicated that B cells were the main target cells in IM. The average viral loads in B cells at day 7 and day 14 were 8.35 × 10^2^ copies/μg DNA (range 5.13 × 10^2^-1.75 × 10^3^ copies/μg DNA) and 6.88 × 10^2^ copies/μg DNA (range 3.7 × 10^2^-1.2 × 10^3^ copies/μg DNA), respectively. Virus loads decreased in B cells more slowly than in plasma (Fig. 1b). The viral loads in plasma and B cells of control group subjects were undetectable.

### Kinetic analysis of expression of EBV-miRNAs in plasma and B cells

**Fig. 2:**
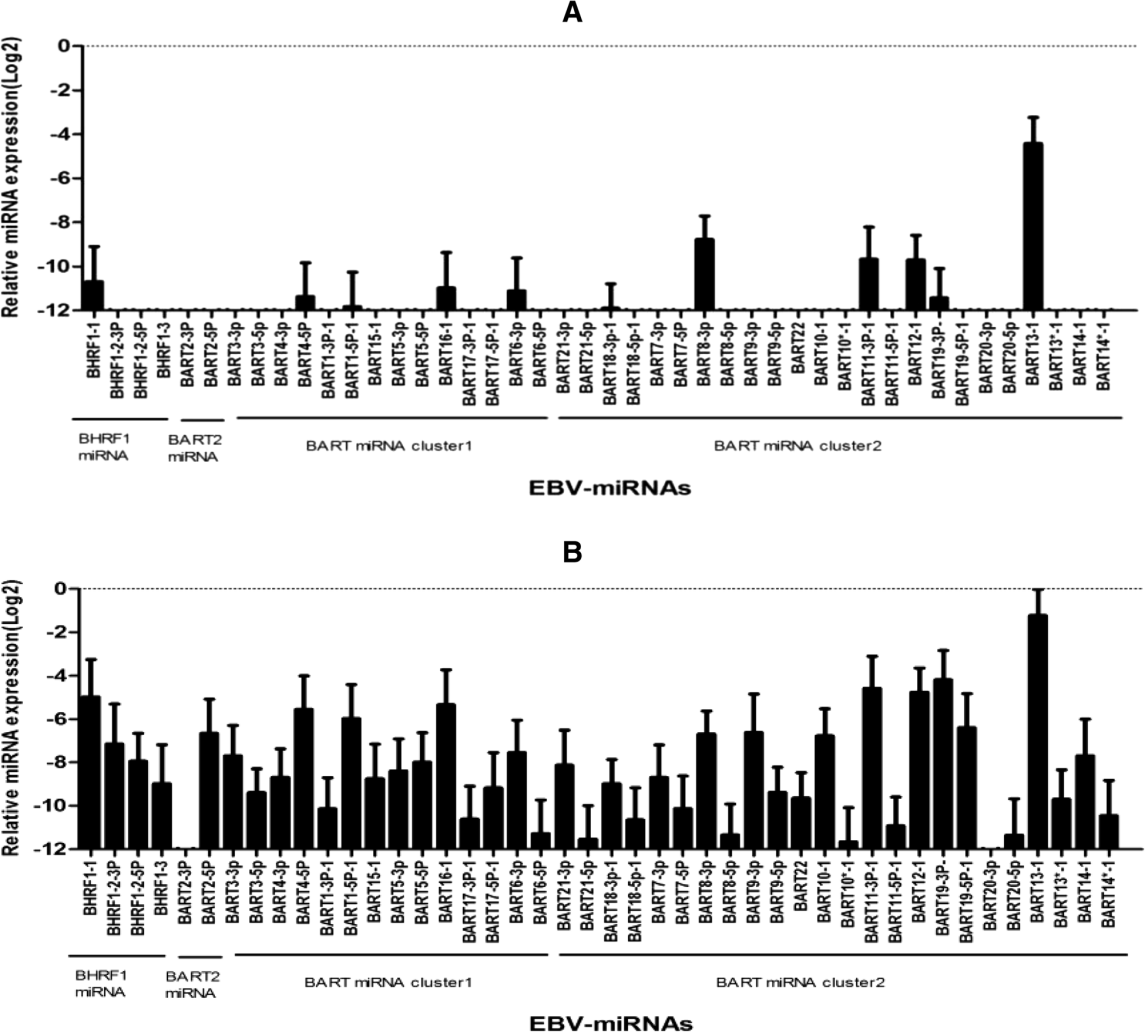
The expression profile of EBV-miRNAs in plasma. **a** The expression profile of EBV-miRNAs in plasma of control group; **b** The expression profile of EBV-miRNAs in plasma of patients with IM at day 0

**Fig. 3:**
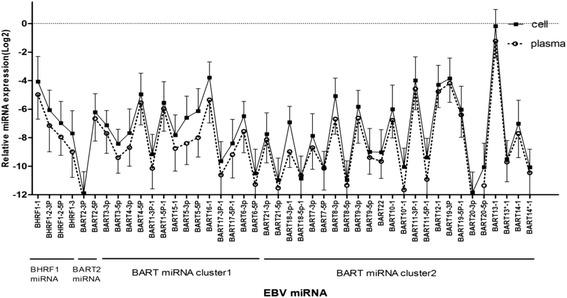
Comparison of the expression profile of EBV-miRNAs in B cells and plasma in patients with IM. All the data were are shown as mean ± standard deviation for the samples

**Fig. 4:**
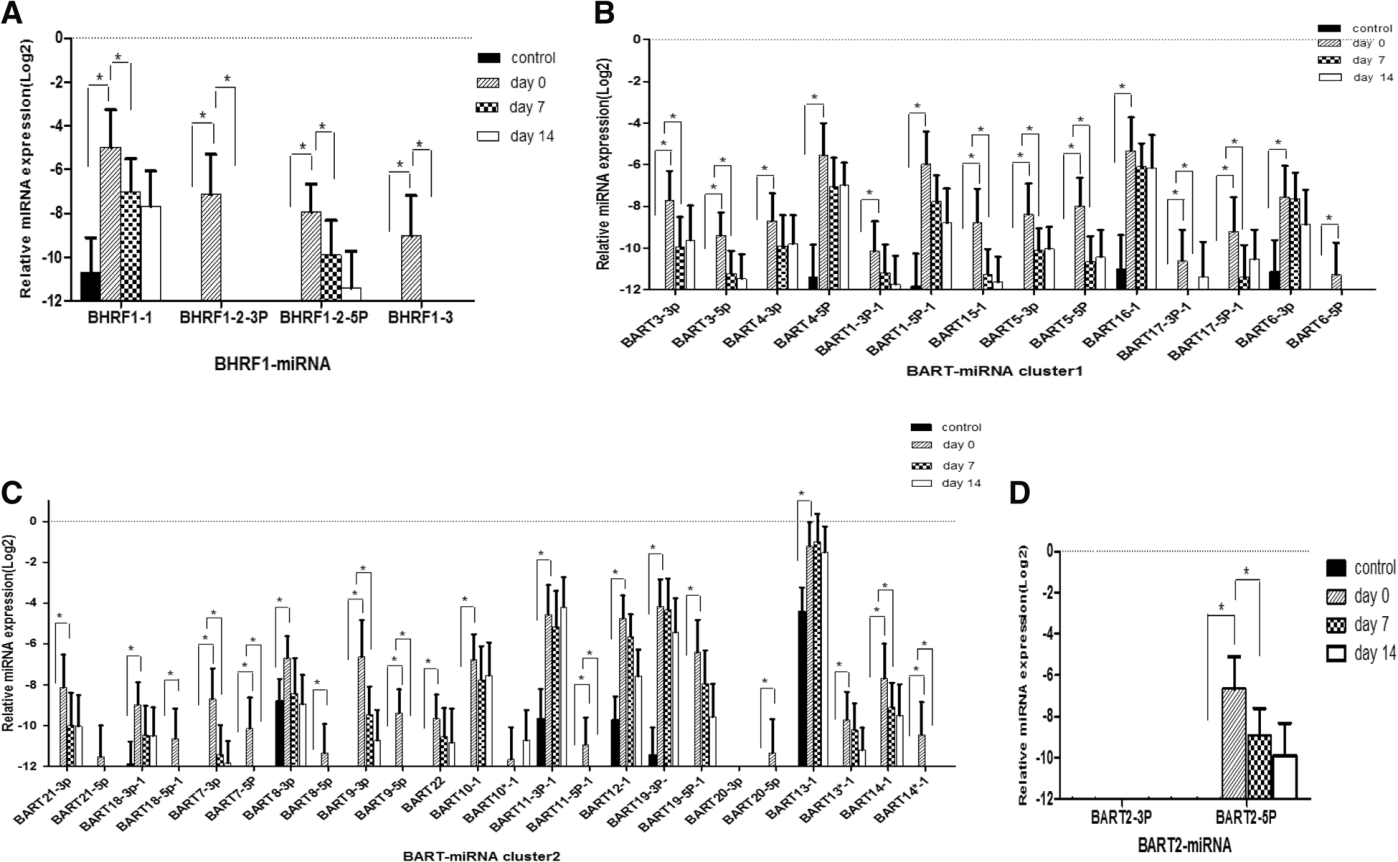
Comparison of the expression profile of EBV-miRNAs in plasma of patients at day 0 (n = 15), day 7 (n = 15), and day 14 (n = 15), and healthy controls group (n = 15). Data represents the mean ± standard deviation for the samples. **a** Comparison of the miR-BHRF1 cluster among the four groups. **b** Comparison of the miR-BART cluster1 among the four groups. **c** Comparison of the miR-BART cluster2 among the four groups. **d** Comparison of BART2-miRNA among the four groups. *P < 0.05 was considered statistically significant

Forty four EBV-miRNAs were quantified by real-time RT-PCR in plasma and B cells in IM patients at days 0, 7, and 14 and healthy control group. Only 11 EBV-miRNAs were detected in the control group (Fig. 2a). As B cells were also analyzed in the control group, the viral loads were less than 5 × 10^2^ copies/μg. At day 0 of patients with IM, nearly all of the EBV-miRNAs expressed in high level except miR-BART2-3P, miR-BART10*-1 and miR-BART20-3p (Fig. 2b). The higher levels of EBV-miRNAs in plasma were miR-BHRF1-1, 1-2-3P, miR-BART13-1, 19-3p, 11-3P, 12–1, 16–1, and 4-5P. The expression profiles of EBV-miRNAs of B cells were similar to those of plasma (Fig. 3). Compared with control group, all the miRNAs except miR-BART2-3P, 20-3p, and 21-5p increased and were significantly different at day 0 (Fig. 4). The expression profile of most EBV-miRNAs in plasma decreased at day 7 and day 14, especially the miR-BHRF1 cluster. At day 7 and day 14, miR-BHRF1-2-3P, 1–3, miR-BART17-3p-1, 6-5p, 21-5p, 18-5p-1, 7-5p, 8-5p, 9-5p, 11-5p-1, 20-5p, and 14*-1 were not detected. Additionally the other EBV-miRNAs were expressed at lower levels at day 14 than day 7. Comparing the expression profile of EBV-miRNAs among different times showed that miR-BHRF1-1, 1-2-3P, 1-2-5P, 1–3, miR-BART3-3P, 3-5P, 5-3P, 5-5P, 15-1(15), 17-3P, 17-5P, 7-3P, 7-5p, 9-3P, 9-5p, 11-5P, 14-3p, 14-5P, and 2-5P were significantly different (Fig. 4). The dynamic changes of expression profiles of EBV-miRNAs in B cells were similar to those of in plasma at day 7 and day 14.

With time progression, the levels of most EBV-miRNAs and viral loads in plasma and B cells both decreased, but the former decreased at a slower rate. Analyzing the correlation of viral loads and EBV-miRNAs in plasma, we found that there was a significant positive correlation between viral loads and some EBV-miRNAs (miR-BHRF1-2-3p, miR-BHRF1-2-5p, miR-BHRF 1–3, miR-BART2-5p, miR-BART3-5p, miR-BART5-5p, miR-BART17-3p, miR-BART6-3p, miR-BART6-5p, miR-BART21-5p, miR-BART7-3p, miR-BART7-5p, miR-BART8-5p, miR-BART12-1, miR-BART19-3p, miR-BART20-5p, miR-BART13*-1 and miR-BART14*-1; 0.85 ≤ r ≤ 0.99, *P* < 0.05).

### Human immunopathology and cell differentiation associated miRNAs in B cells

**Fig. 5:**
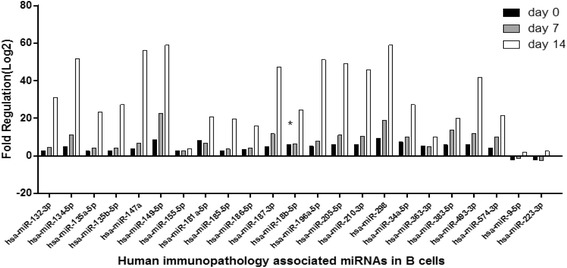
The fold change of top 23 gradually up-regulated and down-regulated cellular immunopathology-associated miRNAs (>2.0 fold) in B cells. Comparing the patients at day 0 (n = 15), day 7 (n = 15), and day 14 (n = 15) with healthy control group (n = 15). *P < 0.05 was considered statistically significant

**Fig. 6:**
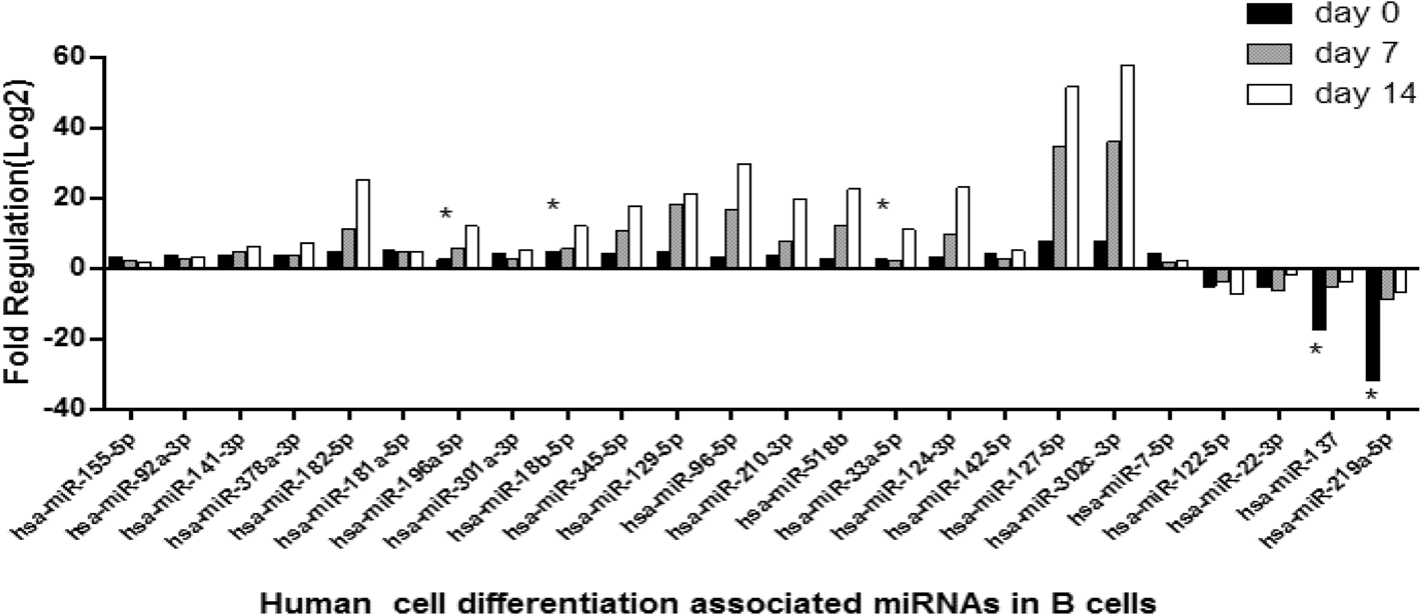
The fold change of top 23 gradually up-regulated and down-regulated cellular differentiation-associated miRNAs (>2.0 fold) in B cells. Comparing the patients at day 0 (n = 15), day 7 (n = 15), and day 14 (n = 15) with healthy control group (n = 15). *P < 0.05 was considered statistically significant

To investigate the dynamic changes of cellular miRNAs in primary infection in B cells, we profiled the expression of 84 immunopathology and 84 cell differentiation-associated miRNAs in B cells of IM patients at days 0, 7, and 14 and control group respectively. Forty cellular miRNAs of day 0 were up-regulated more than 2-fold compared to the control group, for example hsa-miR-134-5p, −18b-5p, −34a-5p, and -196a-5p, −155-5p, −181a-5p, −142-5p; while 6 cellular miRNAs, including hsa-miR-9-5p, −223-3p, −122-5p, −22-3p, −137, and -219a-5p were down-regulated more than 2-fold (See Figs. 5 and 6). Moreover, nearly all of these human miRNAs increased at day 7 and day 14, including hsa-miR-134-5p, −18b-5p, −34a-5p, and -196a-5p (See Figs. 5 and 6). Additionally, hsa-miR-146a was up-regulated later more than 2-fold. These cellular miRNAs were likely associated with immunopathology and differentiation which may play roles in the transition from resting to proliferating B cells. The results showed that EBV loads had negative correlation with hsa-miR-137 (r = −0.99, *P* < 0.05) and hsa-miR-219a-5p of B cells (r = −0.97, *P* < 0.05).

### Human immunopathology cellular miRNAs in CD8+ T cells

**Fig. 7:**
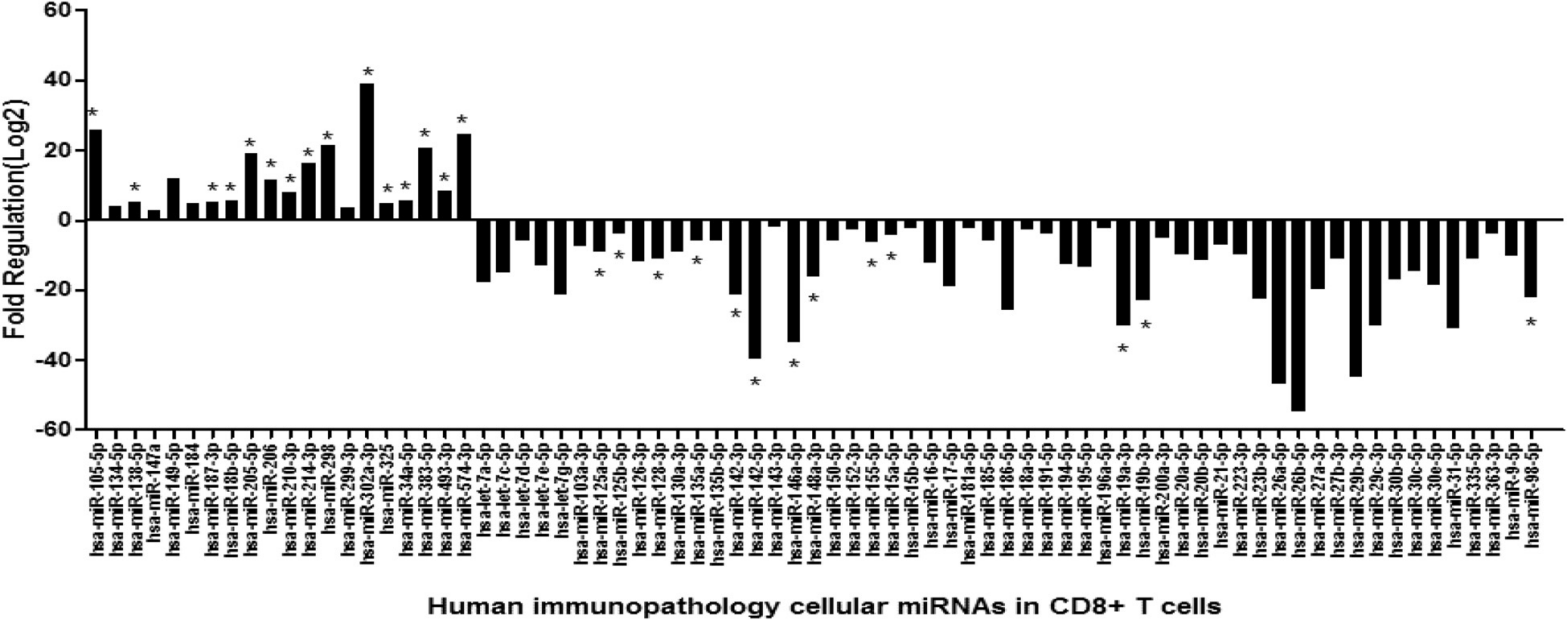
The fold change of top 75 gradually up-regulated and down-regulated cellular immunopathology-associated miRNAs in CD8+ T cells at day 0 of patients with IM (n = 15) compared with control group (n = 15). *P < 0.05 was considered statistically significant

**Fig. 8:**
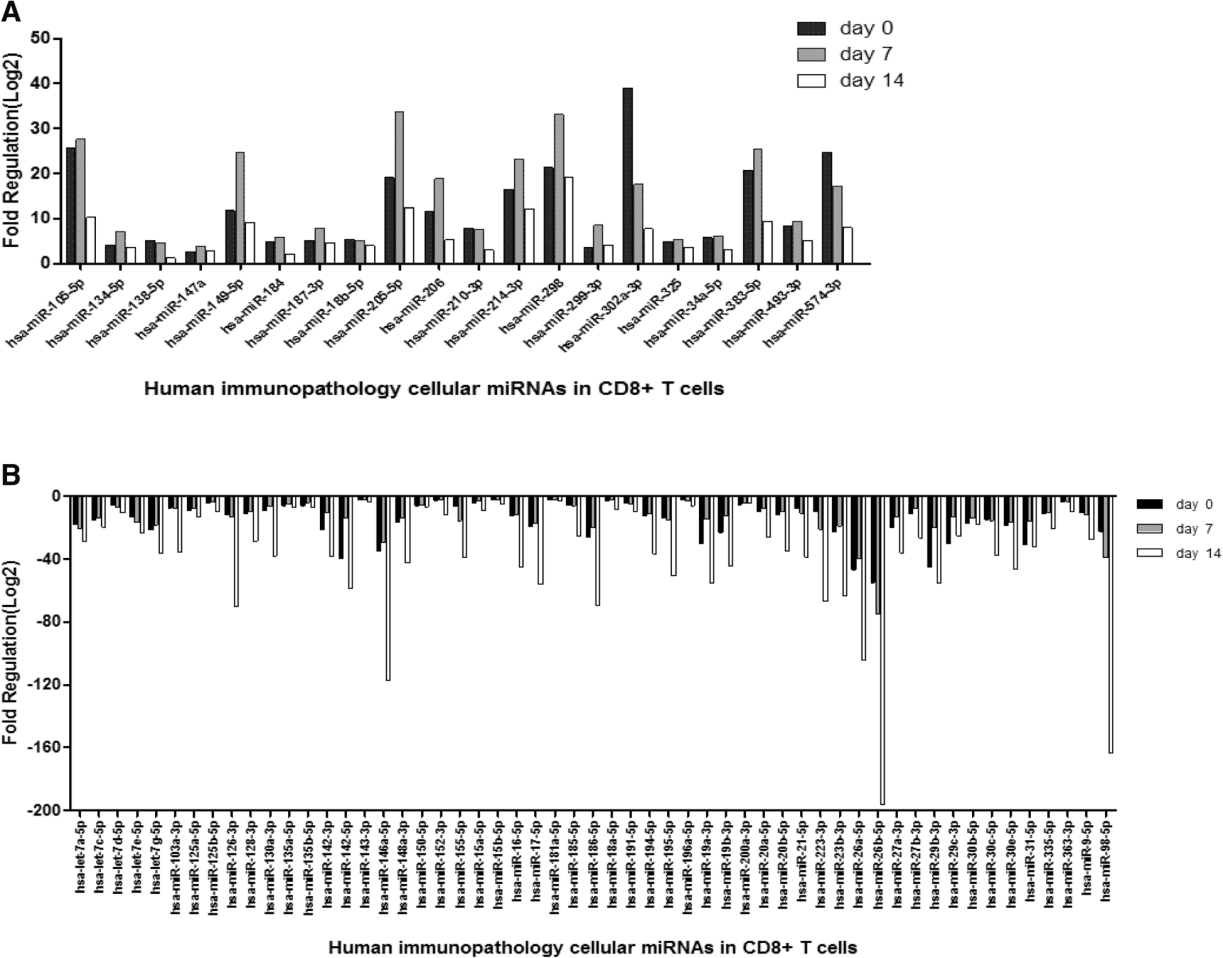
The fold change of immunopathology-associated miRNAs in CD8+ T cells among patients at day 0 (n = 15), day 7 (n = 15), and day 14 (n = 15) compared with healthy control group (n = 15). **a** The up-regulated cellular miRNAs at different times. **b** The down-regulated cellular miRNAs at different times

Eighty four human immunopathology associated miRNAs in CD8+ T cells were detected in patients with IM at days 0, 7, and 14 and control group respectively. At day 0, 55 miRNAs were down-regulated, including hsa-let-7a-5p, hsa-miR-9-5p, −223-3p, −135a-5p, −135b-5p, −142-3p, −142-5p, 146a-5p, and −155-5p, while 20 miRNAs were up-regulated, including hsa-miR-134-5p, −147a, −149-5p, −18b-5p, −205-5p, hsa-miR-206, −210-3p, and -34a-5p (See Fig. 7). At day 7 and day 14, most of the cellular miRNAs of CD8 + T cells, including hsa-let-7a-5p, −142-3p, −142-5p, and −155-5p, decreased with time (See Fig. 8). The results showed that EBV loads had positive correlation with hsa-miR-302a-3p of CD 8 + T cells (r = 0.91, *P* < 0.05).

### Correlation of the levels of EBV-miRNAs and cellular miRNAs

Comparing the relationship between cellular miRNAs and EBV-miRNAs, we determined that hsa-miR-18b-5p of CD8+ T cells exhibited positive correlation with BHRF1-2-5P, BART2-5P, 4-3p, 4-5p, 16–1, 6-3p, 22, 12–1, 19-3P, 19-5P, 13–1, and 13*-1 (0.96 ≤ r ≤ 0.99, *P* < 0.05). Hsa-miR-155-5p of B cells had positive correlation with BHRF1-1, 1-2-5P, BART2-5P, 3-3p, 4-3p, 4-5p, 1-3P-1, 1-5P-1, 22, 12–1, 13–1, and 13*-1 (0.97 ≤ r ≤ 0.99, *P* < 0.05). Additionally hsa-miR-181a-5p of B cells had positive correlation with BART4-3p, 4-5P, 16–1, 22, 10–1, 12–1, 19-3P, 19-5P, 20-3p, 13–1, and 13*-1 (0.97 ≤ r ≤ 0.99, *P* < 0.05).

## Discussion

This is the first study that describes the dynamic expression profile of viral and cellular miRNAs in IM with primary EBV infection. In the early phase, nearly all of the EBV-miRNAs were expressed at high levels, especially miR-BHRF1-1, 1-2-3P, and miR-BART13-1. Cai et al. [4] previously demonstrated that both BHRF1 and BART miRNAs were expressed during primary EBV infection, but BHRF1 miRNAs were expressed at higher levels than BART miRNAs, which is consistent with the results of this study. The increased levels of BHRF1 and BART-derived miRNAs during acute phase of infection were likely due to their location. Feederle et al. [16] showed that miR-BHRF1-2 and −3 are located in the 3’UTR of the early lytic protein BHRF1. Using EBV-infected cells in vitro, Amoroso et al. [17] showed that BHRF1-2 and 1–3 miRNAs increased as early as 24 h after the onset of lytic infection, but BHRF1-1 increased until 48 h or later. However, in our study, miR-BHRF1-1 increased higher than miR-BHRF1-2 and 1–3, which might be due to the patients being in different phases of infection. Seto et al. [18] demonstrated that elevated BHRF1 cluster of miRNAs inhibited apoptosis of infected B cells during primary EBV infection. Therefore, elevated expression of miR-BHRF1 in IM as shown in our study indicated that it might contribute to proliferation of infected B cells. Xia et al. [6] have shown that miR-BHRF1-3 can target CXCL11 which contributes to B cell transformation and immune evasion by modulating host cytokine networks.

Over time, BHRF1 miRNAs decreased or even undetected, but miR-BART13-1, 11-3p-1, 16–1, and 19-3p were expressed at relatively high rates. Thus, in later phase of infection, BART miRNAs may play important roles in EBV infection maintenance. Cameron et al. [14] showed that most of the BART miRNAs were essential for B cell transformation. Riley et al. [19] showed that BART13-3p could target CAPRIN2, a Wnt signaling enhancer whose over-expression enhances apoptosis, and can maintain EBV persistence during latent infection in B cells. BART16 and BART1-3p targeted caspase-3 that block or inhibit apoptosis [20]. Ross [21] demonstrated that miR-BART11-5p could target early B cell factor (EBF1) which plays roles in B cell transformation. At different times of infection, the profile patterns of EBV-miRNAs in plasma were similar in EBV-infected B cells in our study, as was previously demonstrated by a previous study [22].

In this study, EBV DNAs were not detected in plasma at day 14, yet some EBV-miRNAs were detected at the same time-point. A previous study [23] demonstrated that mature EBV-miRNAs are secreted by EBV-infected B cells through exosomes. And exosomes can protect EBV-miRNAs from degradation by RNases. This potentially explains why some patients with higher EBV-miRNAs did not have measurable EBV DNA loads in plasma. This also implied that EBV-miRNAs were more stable, and it could be used as potential markers for diagnosis and treatment for EBV associated diseases. Kawano et al. [24] pointed out that the levels of miR-BART2-5p, 13, and 15 in plasma correlated with the severity and prognosis of CAEBV. Zhou et al. [25] found that levels of several EBV-miRNAs (miR-BART3-3p, 3-5p, 1-3p, 1-5p, 15–1, 5-3p, and 16–1 et al.) in Epstein-Barr virus-associated hemophagocytic lymphohistiocytosis (EBV-HLH) were higher than in IM and healthy controls, and suggested that plasma miR-BART16-1 could be a potential biomarker for monitoring EBV-HLH progression.

Besides encoding its own viral miRNAs, EBV infection also affects the expression of cellular miRNAs which are able to regulate the proliferation and differentiation of B and T cells [8]. Our study first investigated the dynamic expression of cellular miRNAs in B cells and CD8 + T cells of IM. In the early time-point after EBV infection, there was dramatic up-regulation of immunopathology and differentiation-associated cellular miRNAs of B cells, such as miR-155-5p, −34a-5p, and -18b-5p, −210-3p, −301a-3p, −302c-3p, −miR-7-5p, −181a-5p, and −142-5p. Cameron et al. [13] also point out that the expression of has-miR-155 and miR-34a in type III EBV latency cells was higher. With time, the expressions of nearly all of these cellular miRNAs increased, and were associated with immunopathology and differentiation which may play roles in the transition from resting to proliferating B cells. Forte et al. [26] reported that over-expression of miR-34a in EBV-driven B cells contributed to the proliferation of these B cells. Linnstaedt et al. [27] showed that miR-155 played a role in B cell development and differentiation and was up-regulated in various EBV associated B cell lymphomas. Zhang et al. [28] showed that over-expression of miR-181a facilitated B cell differentiation. Therefore, in our study, the expression of miR-34a, miR-155, and miR-181a were elevated in primary EBV infection which may contribute to B cell differentiation and proliferation. Zhang et al. [28] also found that the expression of miR-30 family and miR-9 were elevated in GC B cells and these miRNAs could target B lymphocyte-induced maturation protein 1 (BLIMP1), which is a gene important for B cell differentiation. However in our study, has-miR-9 decreased in peripheral B cells of IM which may be different from GC B cells.

However, in CD8 + T cells, various immunopathology-associated cellular miRNAs were down-regulated. In our study, has-miR-142, −223, −29c-3p, −181a, −200a-3p, −195-5p, let-7a-5p, −7c-5p, −7 g-5p, and −155-5p were down-regulated. Previous studies [29, 30] also demonstrated that the levels of has-miR-29, −142, −let-7, and -181a in effector CD8+ T cells were significantly down regulated. Over time, most of the cellular miRNAs decreased. Previous study [31] showed that miR-181a could reduce the numbers of circulating T lymphocytes and affect the differentiation of T lymphocytes, as well as disrupt distribution of CD4+ and CD8+ T cells. In our study, we found that the expression of miR-181a was down-regulated at the early phase of infection, and was different from B cells, which may contribute to the expansion of CD8+ T cells. In addition to the modulation of T lymphocyte differentiation, miRNAs have been demonstrated to regulate T cell activation. A previous study [32] showed that miR-181a could alter the T cell threshold and sensitivity to antigens. Some studies [27] also demonstrated that over-expression of miR-142-3p and miR-142-5p in T cells may be key mediators of cellular signaling and can regulate immune function. This could protect infected B cells from being killed and enter into latent infection.

Additionally, our study showed that hsa-miR-18b-5p of CD8+ T cells had positive correlation with BHRF1-2-5P and BART2-5P. Hsa-miR-155-5p of B cells had positive correlation with BHRF1-1, while hsa-miR-181a-5p of B cells had positive correlation with BART16-1 and BART22. Further research on the roles of EBV-miRNAs and cellular miRNAs will provide new insights into the complex gene regulation network.

## Conclusions

Viral and cellular miRNAs play important roles in the pathogenesis of diseases associated with EBV infection [19]. The expression profile of viral and cellular miRNAs in IM with primary EBV infection were first described in this study. These results provide the basis of investigating the pathogenic mechanism of EBV-related diseases and bring new insights into the diagnosis and treatment these diseases. However, the potential gene targets of EBV and cellular miRNAs need to be identified in future studies.

## Methods

### Study subjects

Fifteen patients (8 males and 7 females) with IM, aged from 1 year and 10 months old to 11 years old (median age, 5 years and 4 months old), were enrolled in this study. IM was defined according to the following previously proposed criteria [33]: (1) presence of at least three of the following clinical manifestations: fever, pharyngitis, cervical lymphadenopathy, hepatomegaly or splenomegaly; (2) serologic profile of primary EBV infection: IgM to EBV viral capsid antigen (VCA-IgM) and IgG to EBV capsid antigen (VCA-IgG) were positive, with absence of an antibody to Epstein-Barr nuclear antigen (EBNA). Within the patient group, 2 mL peripheral blood of each patient were obtained at three different time points, including days 0, 7, and 14. Day 0 was defined as the admission day for IM patients, while 7 days later and 14 days later were defined as day 7 and day 14 respectively. Fifteen healthy children who were seropositive for EBV were enrolled as control group.

The study design was in accordance with the Helsinki Declaration and was approved by the Beijing Children’s Hospital Ethics Committee. Informed consent was obtained from the parents or legal guardians of the pediatric participants.

### Sample collection and cell isolation

Two mL peripheral blood sample of patients was collected into an EDTA-containing tube, and centrifuged to isolate plasma and peripheral blood mononuclear cells (PBMCs) using the Lymphocyte Separation Medium (Sigma, MO, USA). Then the PBMCs were fractionated into CD8+, CD19+, CD4+, and CD56+ cells by means of IMag (BD Biosciences). The purity of each PBMC subpopulation in our system was typically >95 % as assessed by flow cytometry analysis.

### Quantification of viral load in plasma and determination of EBV-infected cells

EBV DNA was extracted from 200 μL of plasma using QIAamp® MinElute® Virus Spin Kit and from four types of lymphocytes (1 × 10^5^ cells) using a QIAmp DNA Micro Kit (Qiagen) following the manufacturer’s instructions. The viral loads were detected by LightCycler480® real-time quantitative PCR (qPCR) using a commercial EBV DNA Quantitative kit (Daan, Guangzhou, China) and viral load of ≥ 5 × 10^2^ particles was considered positive. Viral loads in plasma and cell samples were respectively expressed as copies per milliliter and copies per microgram of DNA. The target cells showed the highest viral loads.

### RNA extraction and detection of EBV miRNA from plasma and B cells by quantitative real-time PCR (qPCR)

Total RNA was carefully extracted from 200 μL of plasma or 1 × 10^5^ B cells of patients with IM and control subjects by using miRNeasy serum/plasma kit (Qiagen) and miRNeasy mini kit (Qiagen) respectively according to the manufacturer’s protocol. In plasma samples, *C. elegans* miR-39 miRNA mimic (Qiagen) was “spiked-in” at the early stage of RNA extraction as a mimic reference. The miScript II RT Kit (Qiagen) was used to synthesize cDNA. Then a miScript SYBR Green PCR kit (Qiagen) was used for the relative quantitation of 44 EBV-miRNAs according to the manufacturer’s protocol and the miScript primers specific to the corresponding mature sequence obtained from miRBase (www.miRBase.org), including four miR-BHRF1 (miR-BHRF1-1, 1-2-3P, 1-2-5P, 1–3), 14 miR-BART cluster 1(miR-BART1-3P, 1-5P, 3-3P, 3-5P, 4-3P, 4-5P, 5-3P, 5-5P, 6-3P, 6-5P, 15-1(15), 16-1(16), 17-3P, 17-5P), 24 miR-BART cluster 2(7-3P, 7-5p, 8-3P, 8-5p, 9-3P, 9-5p, 10-1(10-3P), 10*-1(10-5P), 11-3p, 11-5P, 12-1(12), 13-1(13-3P), 13*-1(13-5P), 14-3p, 14-5P, 18-3p, 18-5p, 19-3p, 19-5p, 20-3p, 20-5p, 21-3p, 21-5p, 22) and two miR-BART2 (miR-BART2-3P, 2-5P). Amplification reactions then were performed by the LightCycler480 System (Roche Diagnosis, Mannheim, Germany) under the following conditions; 15 min initial hold at 95 °C, 40 cycles of 94 °C for 15 s, 55 °C for 30 s, 70 for 30 s. Data from quantitative RT-PCR were analyzed using the comparative threshold cycle (C_t_) method. For all plasma samples, *C. elegans* miR-39 (Qiagen) was used as the endogenous reference, but for all the samples of cells, cell-derived U6 snRNA (Qiagen) was used as the endogenous reference to normalize the relative expression of EBV-miRNAs. ΔC_t_ was given as the C_t_ of difference between the target miRNA and the endogenous control. The amount of miRNAs was given by the arithmetic formula 2^ΔCt^. Note that relative expression levels are by definition values without units.

### Detection of human cellular miRNA from B cells and CD8 + T cells

Total RNA was extracted from 1 × 10^5^ B and CD8+ T cells from the patients and the controls by a miRNeasy mini kit (Qiagen) according to the manufacturer’s protocol. A real time quantitative PCR assay was performed as described above. Eighty four human immunopathology (Table 1) associated miRNAs in B cells and CD8+ T cells were analyzed using Human Immunopathology miScript miRNA PCR Array (QIAGEN, MIHS-104Z). Eighty four human cell differentiation (Table 2) associated miRNAs in B cells were analyzed using Cell Differentiation & Development miRNA PCR Array (QIAGEN, MIHS-103Z). U6 was used as the endogenous reference. Data analyses were performed with the web-based software package for the RT2 Profiler PCR Array Data Analysis (http://www.Sabiosciences.com/pcr/arrayanalysis.php).

**Table 1:** 84 Human immunopathology associated miRNAs

hsa-let-7a-5p	hsa-let-7c-5p	hsa-let-7d-5p	hsa-let-7e-5p
hsa-let-7g-5p	hsa-miR-103a-3p	hsa-miR-105-5p	hsa-miR-125a-5p
hsa-miR-125b-5p	hsa-miR-126-3p	hsa-miR-128-3p	hsa-miR-130a-3p
hsa-miR-132-3p	hsa-miR-134-5p	hsa-miR-135a-5p	hsa-miR-135b-5p,
hsa-miR-138-5p	hsa-miR-142-5p	hsa-miR-142-3p	hsa-miR-143-3p
hsa-miR-145-5p	hsa-miR-146a-5p	hsa-miR-147a	hsa-miR-148a-3p
hsa-miR-149-5p	hsa-miR-150-5p	hsa-miR-152-3p	hsa-miR-155-5p
hsa-miR-15a-5p	hsa-miR-15b-5p	hsa-miR-16-5p	hsa-miR-17-5p
hsa-miR-181a-5p	hsa-miR-182a-5p	hsa-miR-183-5p	hsa-miR-184
hsa-miR-185a-5p	mi-186a-5p	hsa-miR-187-3p	hsa-miR-18a-5p
hsa-miR-18b-5p	hsa-miR-191-5p	hsa-miR-194-5p	hsa-miR-195-5p
hsa-miR-196a-5p	hsa-miR-19a-3p	hsa-miR-19b-3p	hsa-miR-200a-3p
hsa-miR-203a-3p	hsa-miR-205-5p	hsa-miR-206	hsa-miR-20a-5p
hsa-miR-20b-5p	hsa-miR-21-5p	hsa-miR-210-3p	hsa-miR-214-3p
hsa-miR-223-3p	hsa-miR-23b-3p	hsa-miR-26a-5p	hsa-miR-26b-5p
hsa-miR-27a-3p	hsa-miR-27b-3p	hsa-miR-298	hsa-miR-299-3p
hsa-miR-29b-3p	hsa-miR-29c-3p	hsa-miR-302a-3p	hsa-miR-30b-5p
hsa-miR-30c-5p	hsa-miR-30e-5p	hsa-miR-31-5p	hsa-miR-325
hsa-miR-335-5p	hsa-miR-34a-5p	hsa-miR-363-3p	hsa-miR-379-5p
hsa-miR-383-5p	hsa-miR-409-3p	hsa-miR-451a	hsa-miR-493-3p
hsa-miR-574-3p	hsa-miR-9-5p	hsa-miR-98-5p	hsa-miR-99b-5p

**Table 2:** 84 Human cell differentiation associated miRNAs

hsa-miR-106b-5p	hsa-let-7e-5p	hsa-miR-20b-5p	hsa-miR-125a-5p
hsa-miR-125b-5p	hsa-miR-122-5p	hsa-miR-155-5p	hsa-miR-126-3p
hsa-miR-22-3p	hsa-miR-92a-3p	hsa-miR-141-3p	hsa-miR-378a-3p
hsa-miR-10a-5p	hsa-miR-182-5p	hsa-miR-302a-3p	hsa-miR-93-5p
hsa-miR-1-3p	hsa-miR-181a-5p	hsa-miR-146b-5p	hsa-let-7f-5p
hsa-miR-196a-5p	hsa-miR-301a-3p	hsa-miR-488-3p	hsa-miR-215-5p
hsa-miR-24-3p	hsa-miR-192-5p	hsa-miR-18a-5p	hsa-miR-100-5p
hsa-miR-9-5p	hsa-miR-137	hsa-miR-15a-5p	hsa-miR-134-5p
hsa-miR-103a-3p	hsa-miR-424-5p	hsa-miR-20a-5p	hsa-let-7i-5p
hsa-miR-222-3p	hsa-miR-99a-5p	hsa-miR-206	hsa-miR-195-5p
hsa-miR-132-3p	hsa-miR-16-5p	hsa-miR-23b-3p	hsa-miR-214-3p
hsa-miR-21-5p	hsa-miR-18b-5p	hsa-miR-345-5p	hsa-let-7c-5p
hsa-miR-26a-5p	hsa-miR-101-3p	hsa-miR-17-5p	hsa-miR-129-5p
hsa-miR-96-5p	hsa-miR-183-5p	hsa-miR-210-3p	hsa-miR-223-3p
hsa-let-7a-5p	hsa-miR-518b	hsa-miR-194-5p	hsa-miR-218-5p
hsa-let-7d-5p	hsa-miR-205-5p	hsa-miR-10b-5p	hsa-miR-185-5 p
hsa-miR-133b	hsa-miR-520 g-3p	hsa-miR-33a-5p	hsa-miR-124-3p
hsa-miR-208a-3p	hsa-miR-142-5p	hsa-miR-150-5p	hsa-miR-128-3p
hsa-miR-15b-5p	hsa-miR-130a-3p	hsa-miR-127-5p	hsa-miR-498
hsa-let-7b-5p	hsa-miR-302c-3p	hsa-miR-219a-5p	hsa-let-7g-5p
hsa-miR-375	hsa-miR-7-5p	hsa-miR-146a-5p	hsa-miR-142-3p

Fold-Change (2^(− Delta Delta C_t_)) was the normalized gene expression (2^(− Delta C_t_)) in the test sample divided the normalized gene expression (2^(− Delta C_t_)) in the control sample. Fold-regulation represented fold-change results in a biologically meaningful way. Fold-change values greater than two indicated a positive- or an up-regulation, and the fold-regulation was equal to the fold-change. Fold-change values less than two indicated a negative or down-regulation, and the fold-regulation was the negative inverse of the fold-change. A *p*-value <0.05 was considered to be statistically significant.

### Statistical analysis

Statistical analysis was performed using SPSS 19.0 software (SPSS). The Mann–Whitney *U* test and Wilcoxon signed rank test were used for comparisons of two groups of patients. For comparisons of three groups, the Mann–Whitney *U* test with the Bonferroni correction was used. Pearson correlation coefficient analysis was used to assess the correlation of the expression levels of miRNAs and numbers of EBV DNA copy and lymphocytes. According to the relative expression of EBV-positive miRNAs in the EBV-negative cell line Ramos, 2^−12^ was defined as the cut off value. Differences with *P* values <0.05 were deemed to be statistically significant.

### Availability of supporting data

Beijing Natural Science Foundation

Award number: 7154195
